# Editorial: Eco-evo-devo: an emergent integrative discipline of biology

**DOI:** 10.3389/fcell.2025.1681036

**Published:** 2025-09-18

**Authors:** Miguel Brun Usan, Rodrigo Nunes-da-Fonseca, Kathryn D. Kavanagh, Jacqueline E. Moustakas-Verho, Roland Zimm

**Affiliations:** ^1^ Centro Andaluz de Biologia del Desarrollo (CABD, UPO-CSIC), Seville, Spain; ^2^ Universidade Federal do Rio de Janeiro Instituto de Biodiversidade e Sustentabilidade, Macaé, Brazil; ^3^ Department of Biology, University of Massachusetts Dartmouth, Dartmouth, NS, United States; ^4^ Luonnonvarakeskus, Helsinki, Finland; ^5^ Helsingin yliopisto, Helsinki, Finland; ^6^ Institute of Functional Genomics Lyon, Ecole Normale Superieure de Lyon, Lyon, France; ^7^ Dublin City University, Dublin, Ireland

**Keywords:** evo-devo (evolution and development), developmental and physiological plasticity, holobiont, evolutionary synthesis, theory of biology, developmental bias, life-history, multi-scale causation

**FIGURE 1:**
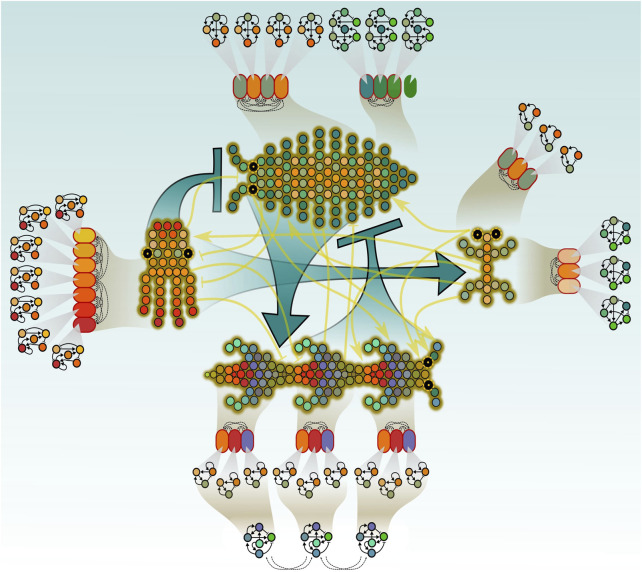
This figure provides a conceptual and metaphorical representation of the aim and scope of the Eco-Evo-Devo research field. From the outer layer to the center, nested networks of genetic, cellular, phenotypic, and ecological interactions generate emergent phenomena and bidirectional causal flows across levels. Eco-Evo-Devo offers a framework to explore this multilevel continuum, revealing hidden regularities, unexpected correlations, and deep organizational principles linking ecology, development, and evolution.

*Eco-evo-devo* (ecological evolutionary developmental biology) has emerged as a highly active field of research, aiming to understand how environmental cues, developmental mechanisms, and evolutionary processes interact to shape phenotypes, morphogenetic patterns, life histories, and biodiversity across multiple scales. Rather than serving as a loose aggregation of diverse research topics and subfields, eco-evo-devo seeks to provide a coherent conceptual framework for exploring causal relationships among developmental, ecological, and evolutionary levels (Figure 1). By doing so, it aspires to be more than the sum of its parts, contributing to the development of a simpler, more elegant, and heuristically powerful biological theory. To illustrate how this integrative approach is taking shape, this Research Topic brings together nine contributions—including empirical studies, conceptual reviews, and opinion pieces, and covering a diverse array of organisms, methodologies and perspectives—that collectively highlight the promise and potential of this emerging discipline.

One central theme is the role of development in shaping plastic phenotypic responses to environmental variation. Beyond the classic reaction-norm-based approaches, which merely establish phenomenological correlations between environmental and phenotypic changes, *eco-evo-devo* aims to provide a causal, mechanistic understanding of how these reaction norms arise during development and evolve over time. Under these premises, Roy et al. present an experimental evolution study in the fruit fly *Drosophila melanogaster*, showing that selection for cold tolerance reduces the plasticity of life-history traits under thermal stress. This finding highlights that, similar to the more widely studied genotype-to-phenotype relationships, development generates complex associations between generative factors - here environmental cues - and phenotypic traits, and that these associations themselves can evolve under sustained environmental selective pressure. Along similar lines, Lofeu et al. demonstrate that the influence of the environment on phenotypic responses extends to the dynamics of development itself. By examining ontogenetic plasticity in the neotropical fish *Astyanax lacustris*, these authors show how temperature modulates developmental responses to different water flow regimes. Together, these studies not only provide evidence for the crucial instructive role of the environment in shaping development and evolutionary potential, but also suggest that such phenomena occur across distantly related taxa and are likely widespread throughout the tree of life. Thus, by challenging the classic view which privileges genetics as the unique central factor in shaping phenotypic evolution, eco-evo-devo provides a new way to understand and test complex interactions between the environment, ontogeny, and inheritance in the study of diversification.

The importance of symbiosis and inter-kingdom communication in physiology, development and evolution is explored from both empirical and conceptual perspectives. Here, the eco-evo-devo framework sheds light on how interactions between different biological agents can generate new layers of complexity and variation. Although the importance of interactions is well established for genes and cells (whose interaction creates and maintains phenotypes), the eco-evo-devo perspective extends the interactive principle of biology to novel levels of organization and new causal mechanisms. For example, developmental processes themselves can be shaped by inter-organismal interactions such as symbiosis and inter-kingdom communication—a subject that is explored in this Research Topic both empirically and conceptually. Mukherjee and Moroz trace the evolution of G-type lysozymes across Metazoa, revealing how these enzymes have been spread by horizontal gene transfer across kingdoms and repeatedly adapted for immune and digestive functions in response to ecological contexts. Scott Gilbert’s opinion article reframes development as a symbiotic process, proposing that organismal identity and morphogenesis are produced through interactions with microbial and environmental partners. These contributions advance the view of organisms as integrated networks of interactions between heterogeneous agents, with eco-evo-devo providing a consistent framework for understanding their mutual development.

Another focus of eco-evo-devo is the role of developmental bias and constraint in directing evolutionary diversification. Stansfield and Parsons review how biases in developmental systems shape adaptive radiations. Their work indicates that variation is not always random or isotropic but influenced by the specific architecture of developmental programs. Singh and Singh explore how gestation length and DNA damage response mechanisms impact life-history traits and correlate with longevity in mammals, emphasizing the developmental foundations of evolutionary transitions in reproductive strategies.

At the intersection of development, morphology and reproductive fitness, two contributions illustrate how developmental processes underpin evolutionary traits. Pintus et al. investigate how Sertoli cell efficiency and sperm size homogeneity influence reproductive potential in the common eland *Taurotragus oryx*, linking cellular development to fertility and sexual selection. Knyazeva and Dyachuk review the neural crest’s role in gland development across vertebrates, highlighting the conserved developmental modules underlying evolutionary innovation in organogenesis, which shows that even macro-evolutionary trends are shaped by conserved developmental mechanisms. Lastly, Moroz and Romanova expand the theoretical foundations of eco-evo-devo, advocating for a reconsideration of functional biodiversity from an eco-evo-devo perspective. Drawing from comparative physiology and evolutionary theory, they argue for a synthesis recognizing the centrality of development in shaping organismal function and ecological adaptation.

These nine articles underscore the broad significance of the eco-evo-devo approach. They demonstrate how developmental processes mediate environmental and evolutionary dynamics, how symbioses, inter-kingdom, and ecological interactions contribute to morphogenesis, and how developmental bias and plasticity influence macroevolutionary patterns. They also suggest future directions of research, including mechanistic studies of developmental-environmental interactions, a broader focus on symbiotic development, and integrative modeling across scales and taxa. Furthermore, they offer a conceptual and empirical strategy to challenge long-held views in order to innovate our fundamental ways of thinking about the dynamics and complexity of nature.

Understanding how organisms respond and evolve in relation to their environments is increasingly important as the planet faces ecological change. Eco-evo-devo provides a comprehensive approach for investigating these dynamics, integrating molecular, developmental, ecological, and evolutionary perspectives. The contributions in this Research Topic reflect the current dynamics of the field with the prospect of establishing a foundation for an integrative biology of the 21st century.

